# Successful Management of Fulminant Immune Checkpoint Inhibitor–Associated Myocarditis Amid Corticosteroid Constraints and Clinical-Biomarker Dissociation

**DOI:** 10.1016/j.jaccas.2025.106787

**Published:** 2026-01-19

**Authors:** Yunting Zhang, Yanwen Fang, Lifang Zhang, Wenjing Wu, Jingang Zheng

**Affiliations:** a Department of Cardiology, China-Japan Friendship Hospital, Beijing, China; b Academy of Medical Sciences & Peking Union Medical College/National Center for Cardiovascular Diseases, Fuwai Hospital, Beijing, China

**Keywords:** cardiac troponin, clinical-biomarker dissociation, corticosteroid-sparing therapy, ICI-associated myocarditis, myocardial remodeling

## Abstract

**Background:**

Immune checkpoint inhibitor (ICI)–associated myocarditis is an uncommon but life-threatening complication. Management is particularly challenging when high-dose corticosteroids are contraindicated and in the presence of clinical-biomarker dissociation.

**Case Summary:**

A 69-year-old man presented with cardiogenic shock and ventricular tachycardia 3 weeks after his first dose of pembrolizumab for lung adenocarcinoma. Despite corticosteroid constraints owing to pulmonary tuberculosis and the complexity of clinical decision-making during tapering with rebound troponin T elevation, the patient achieved favorable long-term survival.

**Discussion:**

This case demonstrates a successful management strategy for fulminant ICI-associated myocarditis under the dual constraints of limited corticosteroid use and persistent biomarker elevation, offering valuable clinical insights.

**Take-Home Messages:**

Rebound elevation of troponin T levels may indicate myocardial remodeling rather than active injury, supporting corticosteroid tapering in clinically stable patients. In patients with ICI-associated myocarditis and contraindications to corticosteroids, early steroid tapering combined with alternative immunosuppressive therapy may still achieve favorable outcomes.

## Graphical Abstract

**Figure:**
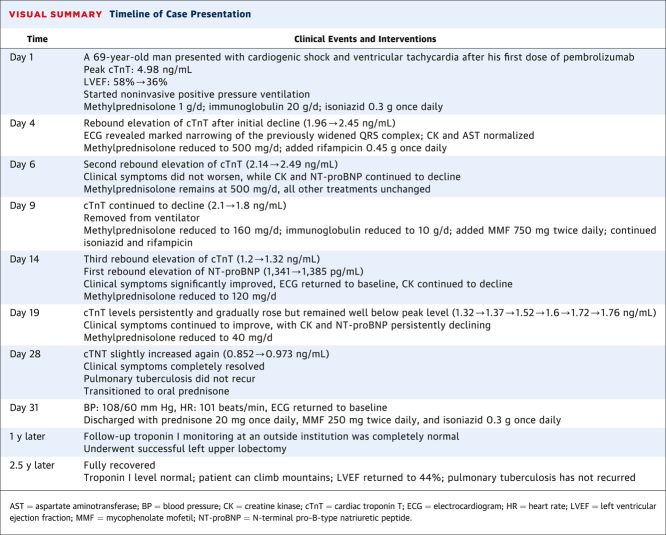

## History of Presentation

A 69-year-old man with a history of heavy smoking, alcohol use, and prior pulmonary tuberculosis was initiated on neoadjuvant chemotherapy with nab-paclitaxel/carboplatin and pembrolizumab for stage IIa (cT2bN0M0) left lung squamous cell carcinoma. Three weeks after treatment initiation, he developed acute dyspnea. On presentation, vital signs showed tachycardia (heart rate: 120 beats/min), hypotension (blood pressure: 92/51 mm Hg), and tachypnea (respiratory rate: 24 breaths/min). Physical examination revealed bibasilar crackles without peripheral edema.

**Take-Home Messages**

• In patients with ICI-associated myocarditis, a rebound elevation in cardiac troponin T levels may suggest myocardial remodeling rather than active injury, supporting a strategy of cautious corticosteroid tapering in those who remain clinically stable.
• In cases of fulminant ICI-associated myocarditis complicated by conditions contraindicating high-dose corticosteroids (such as active pulmonary tuberculosis), multidisciplinary evaluation should be performed to guide early corticosteroid tapering combined with alternative immunosuppressive therapies such as intravenous immunoglobulin or mycophenolate mofetil, which may still enable favorable clinical outcomes.

## Past Medical History

The patient had a history of tobacco use, alcohol consumption, and pulmonary tuberculosis, and was currently maintained on isoniazid therapy. Previous echocardiography demonstrated normal cardiac function.

## Differential Diagnosis

Although the patient's clinical presentation and immune checkpoint inhibitor (ICI) treatment history were highly suggestive of ICI-associated myocarditis, emergent coronary angiography was nevertheless performed to exclude acute coronary syndrome, given the presence of hemodynamic instability, electrical disturbances, and markedly elevated cardiac biomarkers. The angiogram definitively ruled out obstructive coronary artery disease. The patient received chemotherapy with nab-paclitaxel and carboplatin. Although certain antineoplastic agents are known to cause cardiotoxicity,^1^ the risk associated with these 2 agents is very low. Moreover, the clinical course was inconsistent with chemotherapy-induced myocardial injury, thus allowing its exclusion as a potential etiology. Other ICI-related immune-related adverse events, such as myasthenia gravis-like syndrome, were deemed less likely in the absence of corresponding neurological symptoms. Although the patient had a history of pulmonary tuberculosis, infectious myocarditis was considered lower on the differential given the absence of fever, negative blood cultures, and the close temporal link to ICI infusion. The global, rather than regional, pattern of left ventricular (LV) dysfunction on echocardiography argued against Takotsubo cardiomyopathy. A diagnosis of fulminant ICI-associated myocarditis was therefore established according to the Brighton Collaboration criteria, integrating the close temporal association with ICI administration, clinical presentation (including hemodynamic compromise and electrical instability), elevated biomarkers, and the exclusion of alternative etiologies.

## Investigations

Laboratory evaluation revealed markedly elevated biomarkers, notably cardiac troponin T (cTnT), which peaked at 4.98 ng/mL (reference: <0.014 ng/mL), as well as N-terminal pro–B-type natriuretic peptide of 6,119 pg/mL, creatine kinase of 15,275 IU/L, aspartate aminotransferase of 1,509 IU/L, and high-sensitivity C-reactive protein of 37.48 mg/L.

**Figure 1:**
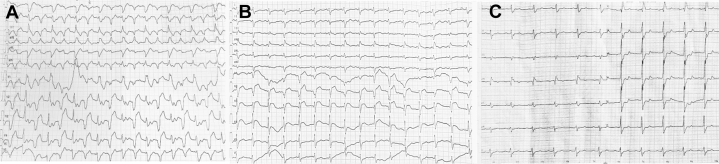
Electrocardiographic Changes During Treatment (A) Electrocardiogram in the cardiac intensive care unit showed ventricular tachycardia. (B) Within 2 days of initiating methylprednisolone at 1 g/d, the electrocardiogram revealed marked narrowing of the previously widened QRS complex. (C) Electrocardiogram at 1 year after discharge.

**Figure 2:**
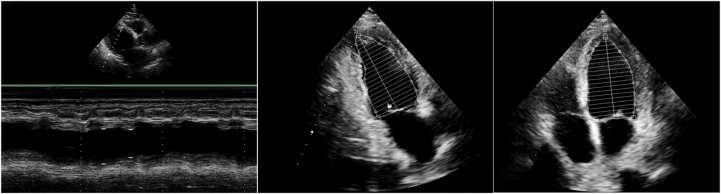
Transthoracic Echocardiography on Admission Transthoracic echocardiography revealed left ventricular dilation and severely reduced systolic function (left ventricular ejection fraction: 36%).

**Figure 3:**
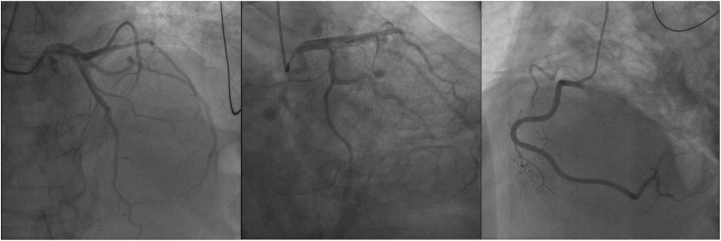
Emergent Coronary Angiography on Admission Coronary angiography excluded obstructive coronary artery disease.

**Figure 4:**
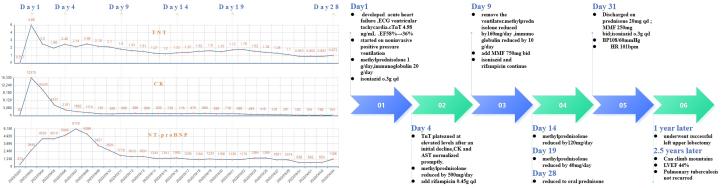
Details of Patient Treatment and Follow-Up Treatment adjustments and follow-up in response to changes in cardiac TnT, CK, and NT-proBNP. AST = aspartate aminotransferase; BP = blood pressure; CK = creatine kinase; ECG = electrocardiogram; HR = heart rate; LVEF = left ventricular ejection fraction; MMF = mycophenolate mofetil; NT-proBNP = N-terminal pro–B-type natriuretic peptide; TnT = troponin T.

Electrocardiography demonstrated ventricular tachycardia, with dynamic electrocardiographic changes illustrated in Figure 1. Transthoracic echocardiography revealed LV dilation and severely reduced systolic function (LV ejection fraction: 36%) (Figure 2). Emergent coronary angiography excluded obstructive coronary artery disease (Figure 3). The changing trends in the patient's cTnT, creatine kinase, and N-terminal pro–B-type natriuretic peptide levels are shown in Figure 4. There was no clinical evidence of other immune-related adverse events.

Classically, both cardiac magnetic resonance imaging and endomyocardial biopsy are recognized components of the diagnostic criteria for ICI-associated myocarditis.^2^ However, endomyocardial biopsy remains underused in clinical practice given its invasive nature and associated procedural risks.^2^ Currently, there is no single gold standard for diagnosing ICI-related myocarditis.^2^ In this case, cardiac magnetic resonance imaging was contraindicated owing to metallic implants, and the patient declined endomyocardial biopsy.

## Management

The patient was immediately initiated on noninvasive positive pressure ventilation and high-dose intravenous methylprednisolone (1 g/d). Given the fulminant presentation of ICI-associated myocarditis and the contraindication to prolonged high-dose corticosteroid therapy owing to pulmonary tuberculosis, second-line therapy with intravenous immunoglobulin (20 g/day) was administered in accordance with American Society of Clinical Oncology guidelines for the management of immune-related adverse events in patients treated with ICIs.^3^ Given his history of tuberculosis, prophylactic isoniazid was initiated alongside intravenous diuretics and low–molecular weight heparin for concomitant lower extremity muscular venous thrombosis.

Within 72 hours, the patient showed remarkable clinical improvement: sinus rhythm was restored, conduction abnormalities improved, hemodynamics stabilized, and dyspnea resolved. LV ejection fraction improved to 38%. Creatine kinase and aspartate aminotransferase levels normalized promptly; however, cTnT plateaued at elevated levels after an initial decline (Figure 4). Given concerns about infection risk with prolonged high-dose corticosteroid therapy, cautious steroid tapering was initiated despite persistent troponin elevation (Figure 4). Mycophenolate mofetil was introduced early during hospitalization as a steroid-sparing agent and was continued for 3 months. The patient remained asymptomatic, without recurrent arrhythmias or heart failure.

## Outcome and Follow-Up

**Figure 5:**
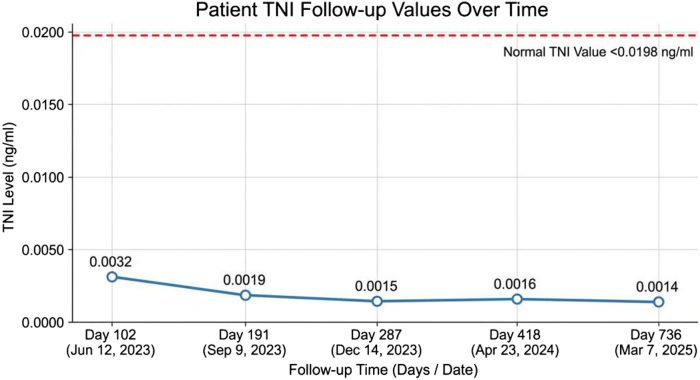
Follow-Up Cardiac Troponin I Values Over Time Serial postdischarge troponin I levels from follow-up at an outside institution. TNI = troponin I.

One year later, the patient underwent successful left upper lobectomy. At the 2-year follow-up, he reported no cardiovascular symptoms, with full unrestricted activity. Echocardiography showed mild LV dilation (LV end-diastolic diameter: 66 mm) and moderately reduced LV ejection fraction (44%).The patient remained on guideline-directed medical therapy, which included sacubitril/valsartan, bisoprolol, spironolactone, and dapagliflozin, with ongoing regular surveillance. At the 2.5-year follow-up, he remained asymptomatic with unrestricted daily activities and showed no signs of tuberculosis recurrence. The specific treatment and follow-up details are shown in Figures 4 and 5.

## Discussion

Among the various immune-related adverse events, cardiovascular manifestations, particularly ICI-associated myocarditis, remain one of the most challenging and potentially life-threatening complications.^4^ This case illustrates the challenging scenario of fulminant ICI-associated myocarditis in a patient with contraindications to prolonged high-dose corticosteroid therapy and persistent biomarker elevation despite clinical improvement. Current guidelines advocate for early administration of high-dose corticosteroids;^5^^,^^6^ nevertheless, the management of such cases requires careful balancing of immunosuppressive needs against infection risks, particularly in patients with comorbidities such as tuberculosis.

The dissociation between clinical improvement and persistent troponin elevation presents a particular challenge in ICI myocarditis management. While cTnT is a highly specific marker of myocardial injury, its persistent elevation may not always indicate active myocardial damage. Emerging evidence indicates that skeletal muscle inflammation—particularly during the repair processes—can lead to the release of cTnT from noncardiac sources. For example, persistent elevation of cTnT without a concomitant rise in cardiac troponin I has been reported in patients with idiopathic inflammatory myopathy.^7^ To investigate this possibility in our case, serial cardiac troponin I measurements were performed and consistently demonstrated elevated levels. In addition, creatine kinase levels normalized and did not exhibit a trajectory paralleling the persistent cTnT elevation, thereby further excluding significant skeletal muscle involvement as the source of troponin release. Clinically, the patient showed substantial improvement in heart failure symptoms, with progressive resolution of atrioventricular block and no recurrence of arrhythmias. Therefore, in the presence of clinical-biomarker dissociation, elevated biomarker levels may reflect myocardial remodeling rather than ongoing injury—a novel insight proposed in the present case report.

When corticosteroid use is limited, alternative immunosuppressive agents become crucial. In this case, intravenous immunoglobulin provided effective immunosuppression without significantly increasing infection risk. Mycophenolate mofetil served as an effective steroid-sparing agent, allowing for earlier corticosteroid tapering while maintaining adequate immunosuppression. Based on this experience, we propose that, under close monitoring, cautious corticosteroid tapering is a feasible strategy in clinically stable patients, which may reduce the risk of steroid-related complications without compromising clinical outcomes.

The successful outcome in this case underscores the importance of multidisciplinary collaboration in managing complex cardio-oncology cases. Input from specialists in cardiology, oncology, infectious diseases, and pulmonology is crucial in formulating an individualized treatment plan that balances oncologic efficacy, cardiovascular risk mitigation, and infection control. Even after successful acute management however, patients with ICI myocarditis require long-term surveillance for cardiac dysfunction, arrhythmias, and potential disease recurrence. No studies have revealed the long-term consequences of ICI-associated myocarditis.^8^ This case demonstrates that with appropriate management, patients can achieve good long-term outcomes despite severe initial presentation.

## Conclusions

This case represents an uncommon example of successful management with sustained favorable outcomes. It reinforces the practice of early and aggressive immunomodulation as the cornerstone of treatment for fulminant ICI-associated myocarditis. Persistent troponin elevation may indicate myocardial remodeling rather than active injury, supporting cautious steroid tapering in clinically stable patients. Multidisciplinary team–guided, individualized treatment strategies that incorporate alternative immunosuppressants and meticulous long-term monitoring can yield successful outcomes, even in high-complexity scenarios. Our experience offers a valuable framework for managing ICI-associated cardiotoxicity when conventional corticosteroid regimens are limited.

## Funding Support and Author Disclosures

The authors have reported that they have no relationships relevant to the contents of this paper to disclose.

**Visual Summary:** Timeline of Case Presentation

Time	Clinical Events and Interventions
Day 1	A 69-year-old man presented with cardiogenic shock and ventricular tachycardia after his first dose of pembrolizumab Peak cTnT: 4.98 ng/mL LVEF: 58%→36% Started noninvasive positive pressure ventilation Methylprednisolone 1 g/d; immunoglobulin 20 g/d; isoniazid 0.3 g once daily
Day 4	Rebound elevation of cTnT after initial decline (1.96→2.45 ng/mL) ECG revealed marked narrowing of the previously widened QRS complex; CK and AST normalized Methylprednisolone reduced to 500 mg/d; added rifampicin 0.45 g once daily
Day 6	Second rebound elevation of cTnT (2.14→2.49 ng/mL) Clinical symptoms did not worsen, while CK and NT-proBNP continued to decline Methylprednisolone remains at 500 mg/d, all other treatments unchanged
Day 9	cTnT continued to decline (2.1→1.8 ng/mL) Removed from ventilator Methylprednisolone reduced to 160 mg/d; immunoglobulin reduced to 10 g/d; added MMF 750 mg twice daily; continued isoniazid and rifampicin
Day 14	Third rebound elevation of cTnT (1.2→1.32 ng/mL) First rebound elevation of NT-proBNP (1,341→1,385 pg/mL) Clinical symptoms significantly improved, ECG returned to baseline, CK continued to decline Methylprednisolone reduced to 120 mg/d
Day 19	cTnT levels persistently and gradually rose but remained well below peak level (1.32→1.37→1.52→1.6→1.72→1.76 ng/mL) Clinical symptoms continued to improve, with CK and NT-proBNP persistently declining Methylprednisolone reduced to 40 mg/d
Day 28	cTNT slightly increased again (0.852→0.973 ng/mL) Clinical symptoms completely resolved Pulmonary tuberculosis did not recur Transitioned to oral prednisone
Day 31	BP: 108/60 mm Hg, HR: 101 beats/min, ECG returned to baseline Discharged with prednisone 20 mg once daily, MMF 250 mg twice daily, and isoniazid 0.3 g once daily
1 y later	Follow-up troponin I monitoring at an outside institution was completely normal Underwent successful left upper lobectomy
2.5 y later	Fully recovered Troponin I level normal; patient can climb mountains; LVEF returned to 44%; pulmonary tuberculosis has not recurred

AST = aspartate aminotransferase; BP = blood pressure; CK = creatine kinase; cTnT = cardiac troponin T; ECG = electrocardiogram; HR = heart rate; LVEF = left ventricular ejection fraction; MMF = mycophenolate mofetil; NT-proBNP = N-terminal pro–B-type natriuretic peptide.
